# *“Kunika women are always sick”*: views from community focus groups on short birth interval (*kunika*) in Bauchi state, northern Nigeria

**DOI:** 10.1186/s12905-020-00970-2

**Published:** 2020-05-24

**Authors:** Umaira Ansari, Juan Pimentel, Khalid Omer, Yagana Gidado, Muhd Chadi Baba, Neil Andersson, Anne Cockcroft

**Affiliations:** 1 Centro de Investigación de Enfermedades Tropicales (CIET), Universidad Autónoma de Guerrero, Calle Pino s/n Colonia El Roble, 39650 Acapulco, Guerrero Mexico; 2 CIET-PRAM, Department of Family Medicine, McGill University, 5858 Chemin de la Cote-des- Neiges, 3rd Floor, Suite 300, Montreal, Quebec H3S 1Z1 Canada; 3 Federation of Muslim Women Association of Nigeria (FOMWAN), Bauchi, Nigeria

**Keywords:** Short birth interval, Child spacing, Northern Nigeria, Hausa, Community, Focus groups, Male involvement

## Abstract

**Background:**

In Northern Nigeria, short birth interval is common. The word *kunika* in the Hausa language describes a woman becoming pregnant before weaning her last child. A sizeable literature confirms an association between short birth interval and adverse perinatal and maternal health outcomes. Yet there are few reported studies about how people view short birth interval and its consequences. In support of culturally safe child spacing in Bauchi State, in North East Nigeria, we explored local perspectives about *kunika* and its consequences*.*

**Methods:**

A qualitative descriptive study included 12 gender-segregated focus groups facilitated by local men and women in six communities from the Toro Local Government Area in Bauchi State. Facilitators conducted the groups in the Hausa language and translated the reports of the discussions into English. After an inductive thematic analysis, the local research team reviewed and agreed the themes in a member-checking exercise.

**Results:**

Some 49 women and 48 men participated in the 12 focus groups, with an average of eight people in each group. All participants were married with ages ranging from 15 to 45 years. They explained their understanding of *kunika*, often in terms of pregnancy while breastfeeding. They described many disadvantages of *kunika*, including health complications for the mother and children, economic consequences, and adverse impact on men’s health and family dynamics. The groups concluded that some people still practise *kunika*, either intentionally (for example, in order to increase family size or because of competition between co-wives) or unintentionally (for example, because of frequent unprotected sex), and explained the roles of men and women in this.

**Conclusion:**

Men and women in our study had a clear understanding of the concept of *kunika*. They recognized many adverse consequences of *kunika* beyond the narrow health concerns reported in quantitative studies. Their highlighted impacts of *kunika* on men’s wellbeing can inform initiatives promoting the role of men in addressing *kunika*.

## Background

The term “child spacing” is sometimes used synonymously with family planning, usually implying use of modern methods of contraception [1, 2]. Women and men at the household level, however, might distinguish between child spacing and family planning; they might favour child spacing and be hostile to the idea of limiting their total number of children [3, 4].

A short interval between successive births can have adverse health consequences irrespective of the total number of children. Short birth interval is linked to adverse perinatal outcomes for the next child, including preterm birth, low birth weight, and small for gestational age babies [5]. In some populations it may be linked to stunting [6]. An analysis of data from demographic and health surveys suggested that almost three million deaths of children below five years of age (about 35% of the total under-five years mortality) in developing countries in 2003 could have been averted if all birth intervals had been at least 36 months [7].

Evidence of adverse maternal health consequences is less clear, but short birth interval might increase the risk of maternal death, anaemia, and uteroplacental bleeding disorders [6, 8]. In 2007, the World Health Organisation recommended an interval of at least 24 months between a live birth and the next pregnancy, thus an interval of at least 33 months between two successive births [9].

Short birth intervals are common in Nigeria, where 62% of women reported an interval between their previous two births of less than 36 months, and 23% an interval of less than 24 months [2]. The mean birth interval in North East Nigeria is lower than the national figure [2]. Short birth interval is a well-recognised concept in Northern Nigeria where the majority of the population is Muslim. *Kunika* is the Hausa term that describes a woman becoming pregnant before weaning her last child. Islamic teaching promotes birth spacing to protect the health of mothers and their children and advises that a woman should not become pregnant before weaning her previous child. The recommended length of breastfeeding is two years, so this means avoiding pregnancy for about two years after a live birth [10].

Several qualitative studies have explored understanding and views about child spacing in Nigeria [4, 11] and elsewhere [3, 12–15], without focusing specifically on views about the consequences of short birth interval. Linked to a project to test the impact of universal home visits to improve maternal and child health in Bauchi State, in North East Nigeria, [16, 17] we sought to develop a culturally safe approach to child spacing for potential inclusion in the home visits. We wanted to explore local views and knowledge about causes of short birth interval (*kunika*) and how to prevent it. We made no assumptions about whether ordinary women and men in Bauchi considered *kunika* to be good or bad. Before exploring the views of local people about causes and prevention of *kunika*, we first sought to understand how they view the phenomenon: is it something positive or something negative and why? The study described here used focus groups to explore the views of men and women in Toro Local Government Area (LGA) of Bauchi State about *kunika* and its consequences. The findings will inform further work in the same communities to systematize local knowledge about causes and prevention of *kunika*.

## Methods

### Setting

Toro LGA in Bauchi State in North Eastern Nigeria borders the restive city of Jos. Most of the Bauchi State population of around five million people are Muslim, and the predominant ethnic group is Hausa. Family size is large, and polygamy is common. Some 57% of women in Bauchi have no education, compared with 36% nationally [18]. Use of contraception is low: just 5% of married women aged 15 to 49 years in Bauchi use any modern method of contraception, and 21% are described as having an unmet need for contraception, compared with figures of 12% using modern methods and 19% with unmet need for contraception nationally [18].

### The focus group guide

We designed the focus group guide to explore several issues. What do women and men in communities of Toro LGA understand by the term *kunika*? What disadvantages and advantages of *kunika* do they perceive? Do they perceive *kunika* overall as a good thing or a bad thing? What factors might push people to practise *kunika*?

A senior female and a senior male member of the Bauchi research team piloted the guide with a female and a male focus group in one community in Toro LGA. We made minor modifications to the guide based on the experience of this pilot. Additional file 1 contains the final version of the guide.

### Focus group implementation

The research team selected six communities to reflect the spread of urban, rural, and remote locations across Toro LGA, excluding communities with security concerns. Team members from Bauchi State, familiar with local customs and traditions, visited each of the six communities to meet the village chief and council, and arranged to conduct the focus groups in a neutral venue, often the school.

A trained woman from the local research team used the guide to facilitate the focus groups of women, with another trained woman taking detailed notes of the discussions. Similarly, two trained men from the local team facilitated and recorded the focus groups of men. The training of the facilitators stressed the importance of remaining neutral, not voicing their own views, and neither endorsing nor challenging views expressed by focus group participants. We did not use voice-recorders during the focus group discussions. The facilitators were graduates and had several years of experience in conducting focus group discussions.

The facilitators conducted a total of 12 focus group discussions: one with men and one with women in each of the six communities. The discussions lasted about an hour and were in the Hausa language. The reporter took detailed notes in Hausa during each group discussion, including some verbatim quotes, and the facilitator and reporter finalised the report on the same day, soon after the discussion ended. They translated the report into English.

### Analysis

Analysis followed the phases of inductive thematic analysis proposed by Braun and Clarke [19]. The lead author, who has experience of field work and data analysis in Nigeria, went through the reports to search for meanings and patterns. She extracted key quotes and points raised in each group. She then grouped points to identify themes and sub-themes and discussed these with another author (AC). In a member-checking exercise [20] the local research team reviewed and finalised the themes.

## Results

In total, 49 women and 48 men participated in 12 focus groups in six communities, with an average of eight people in each group. All the group participants were married, with ages ranging from 15 to 35 years for women, and 25 to 45 years for men. Few of the group members had beyond primary education, and many of the women did not have any formal education.

### Understanding *kunika*

All participants in the focus groups were familiar with the concept of *kunika* and had clear ideas about what it is. They agreed it means having a short interval between two successive births, or giving birth frequently.

*Kunika* means a woman frequently giving birth and the children end up growing together like twins. (Female focus group).

Many people referred to a woman becoming pregnant while lactating, before she has weaned her previous child.

Oh! I think *kunika* is a situation where a child is not properly breastfed. The mother becomes pregnant while the baby is still sucking breast milk. (Female focus group).

An infant at hand and an infant in the womb at the same time. This is *kunika* - getting pregnant before weaning. (Male focus group).

Some mentioned the failure of lactational amenorrhoea as a cause of *kunika*.

*Kunika* is when a woman is menstruating after delivery, and suddenly she gets pregnant. (Female focus group).

A few defined *kunika* in terms of months or years between births.

*Kunika* is giving birth to two children within two years. (Everybody started laughing) two years, indeed! (Female focus group).

*Kunika* is having another pregnancy after delivery by one, two, three or four months. (Male focus group).

Others defined *kunika* by reference to the development of the existing child.

*Kunika* is when the previous child has not yet started walking and the mother becomes pregnant again. (Female focus group).

### Perceived disadvantages of *kunika*

All the groups were clear and vocal that *kunika* was negative, with many disadvantages.

There is nothing good about *kunika* (Female focus group).

You know, anything regretful is not a good thing (Male focus group).

They described disadvantages and dangers of *kunika* for the pregnant woman, the baby, the husband, and the family at large.

#### Health complications for the mother

Participants in all the groups considered that *kunika* is harmful for the mother and leads to medical complications. They referred to the many physical health problems experienced by “*kunika* women”, the local term for women who become pregnant soon after their last delivery.

*Kunika* women are always sick. Sometimes they find it very difficult during delivery. (Female focus group).

Indeed, some women have to undergo c-section because they have complications during delivery. (Female focus group).

When a woman has *kunika* she may even die during delivery because she will lose a lot of blood. (Female focus group).

Whenever a woman gives birth her uterus becomes weak. If she frequently gives birth, it will become weaker and weaker. (Male focus group).

It sometimes causes miscarriage or forces the woman to abort the pregnancy. This may lead to danger for both the child and the mother. (Male focus group).

It leads to losing their life, both mother and the child. (Male focus group).

Group participants also mentioned mental health problems for *kunika* women. They said that *kunika* women are stressed and may become depressed and unable to take care of themselves and their babies.

She will be in a lot of tension, especially when she is watching her child become malnourished because of a lack of breast milk. (Female focus group).

The woman will be depressed, and the child won’t get much attention. (Male focus group).

*Kunika* makes a young girl to look older than her age. (Male focus group).

#### Health complications for the new child

Focus group participants referred to children born after too short a birth interval as “*kunika* children” and agreed that these children face health risks.

*Kunika* children die because of malnutrition. (Female focus group).

A *kunika* baby has frequent diarrhoea, running nose, malaria and other diseases. (Female focus group).

It becomes a calamity. I once heard that a woman, who had experienced frequent *kunika*, was terribly worried about the health of her children. She prayed to God to spare even one of her children, because they were all sick. (Male focus group).

*Kunika* children fall sick more often than non-*kunika* children. They develop deformities. (Male focus group).

#### Health complications for the preceding child

Participants also highlighted health risks for the preceding child, since the mother will have to wean this child early when she becomes pregnant again.

Even though the baby is small, the mother will have to wean him or her early. This can make the baby sick and sometimes it leads to the death of the baby. (Female focus group).

It affects the child’s health because the child is taken away from breast feeding while he is not taking any solid food. Therefore, malnutrition and deficiency are definitely going to occur. (Male focus group).

Apart from early weaning, some people pointed out that the preceding child is neglected and poorly because the mother herself is ill and weak, and unable to look after the child.

Ahh! The pregnant woman will be very weak and the child she is nursing will be weak and malnourished at the same time. (Female focus group).

Definitely, the pregnant woman will neglect her baby. She can’t cope with the stress of pregnancy and taking care of an infant. (Female focus group).

#### Economic impact

Participants explained that pregnancies at short intervals create a big financial burden on the family. With more dependent people to feed on the same income, families may suffer food shortage. Families of *kunika* women also spend more on health care for the pregnant woman and her children, because of the health impacts of *kunika* on women and their children.

Even if the husband is well-to-do, *kunika* will make him poor because he has to make double efforts to take care of the child, the pregnant woman and the unborn child. (Female focus group).

It causes poverty because you will sell all your food stuff to get money so that you can settle the hospital bill. You will become poor, you will not have food to feed the remaining children, and the sick one may die, so it’s really a terrible thing. (Male focus group).

The increased financial burden mainly affects men, who are the main breadwinners. They struggle to keep up with the demands of a rapidly growing family, as well as having to provide money for naming ceremonies of children born in quick succession.

Let me tell you something: even the husband will not be able to take care of his family. *Kunika* causes families to become very large. (Female focus group).

The responsibility of the naming ceremony and other expenses falls on the man. Any time you get *kunika*, you will get sad and frustrated. The man will just hate the woman. (Male focus group).

#### Impact on men’s health

Male focus group participants highlighted how *kunika* may cause mental and even physical health problems for men as well as women.

The man suffers a lot when there is *kunika* in the house. (Male focus group).

The father will be depressed and may end up with hypertension. (Male focus group).

In fact, it reduces the strength of manhood. (Male focus group).

#### Deterioration of family dynamics

Participants frequently expressed that *kunika* creates discord and friction within the family, and they described mechanisms for this. The husband is under stress to provide for a large family. The money is not enough for his growing family and he has to look for an added source of income. He doesn’t take care of his wife and starts neglecting her. The wife, on the other hand, is weak and neglects her children and her house. She is frequently ill and might be depressed. All this creates a toxic environment in the family.

My sister, *kunika* contributes to family conflicts. The husband doesn’t care about the needs of his wife during pregnancy, she is left to suffer and bear the consequences alone. (Female focus group).

It leads to misunderstanding between husband and wife. She may not perform her domestic responsibilities very well because she has a little infant and she is pregnant as well, and her husband still needs her to do the work. You see, there will be a conflict. (Male focus group).

The frequent pregnancies can lead to sexual problems between the man and the woman. The man may lose interest in his frequently pregnant wife. The tired and depressed woman might lose interest in the husband. The husband might force sex with the wife.

The husband loses attention of his wife and the wife also loses attention of her husband. There is no love. (Female focus group).

Which husband will love a pregnant woman when she is heavy, weak and clumsy? (Female focus group).

My sister, all men are dirty, but no husband wants to come close to a dirty or smelling wife. (Female focus group).

It triggers conflict between couples because most times a man forces his wife or insists to have sex with her, and since she is pregnant, she will be upset. (Male focus group).

*Kunika* women are perceived as being lazy and dirty and failing to look after their households properly.

When a woman is doing *kunika*, her problems are too many. She is sick every day and her house is always dirty. (Female focus group).

Your fellow women will be teasing you. They will say: *Kunika* women are lazy and dirty. (Female focus group).

It causes a woman to be dirty with her clothes, and her environment. The child also will not have proper care. (Male focus group).

Children in *kunika* households suffer from lack of food, lack of care, and overcrowding.

With *kunika* it is the children who suffer; they don’t get enough food and care. (Female focus group).

It is not conducive when there are many children: there is no place to lay their heads, sleeping becomes a problem. (Female focus group).

Because the baby is neglected, older children beat up the new baby. (Female focus group).

#### Social taboo

The focus groups noted that *kunika* is not socially accepted in their communities. People make fun of *kunika* women. One reason for this might be that people associate *kunika* with frequent sexual activity, which in a conservative society like Bauchi would be a matter of shame and embarrassment.

Women who do *kunika* are considered as hyper-sexual. Therefore, they are shamed. (Female focus group).

A *kunika* woman is always ashamed of herself. Sometimes she doesn’t even want her family to know that she is doing *kunika*. (Female focus group).

When they see your children, they will say, ‘Hey, look at those *kunika* children'. (Male focus group).

People will look at you, mocking you and the family. (Male focus group).

### Why *kunika* still happens, despite its disadvantages

Asked about advantages of *kunika*, focus group participants said that they did not consider it had advantages at all. The facilitators probed further, asking why it still happens, despite nearly everyone believing it to be a bad thing, with many adverse consequences. The discussion focused on three areas: some people choose *kunika*; *kunika* is an unintentional consequence of frequent sexual activity; and *kunika* is simply the Will of God.

#### Some people choose kunika

Focus group participants identified several reasons why people might choose *kunika*.

##### A desire to have a large family

For some people, having more children translates as having more helping hands in the family. Children help with household chores, and they earn money when they grow up. For some men, a larger family is a source of pride.

It is just for having more children to reduce a workload within the household. (Male focus group).

If a man has more children, they will help him with many activities. (Male focus group).

Men who want to have plenty of children like to do *kunika*. (Female focus group).

Yes, some are even proud to have a large family. (Male focus group).

##### Women wanting to complete their families quickly

Some women practise *kunika* because it helps them complete their families quickly, either at the beginning of their reproductive life or if they marry late.

Some women want to give birth at an early age and rest. (Female focus group).

It is an advantage for a woman to do *kunika* to complete her deliveries quickly otherwise it takes her a long time before she completes her family. (Male focus group).

Some people marry late. *Kunika* is an advantage for them to get more children quickly. (Male focus group).

##### Co-wives competing with each other

Polygamy is usual in Bauchi and focus group participants identified competition between co-wives to have more children as a reason why they might practise *kunika*. Wives with more children are entitled to a larger share of their husband’s property.

Women do *kunika* for competition. They gather a lot of children quickly so that they may benefit from inheritance. (Male focus group).

Co-wives may also compete with each other for their husband’s attention and care. The wife who gives birth in quick succession is seen as the one who has his love and attention.

Some women do *kunika* because they want to ensure that they are loved by their husbands. (Female focus group).

#### Kunika happens unintentionally because of frequent sexual contact

Participants in men’s groups said that *kunika* happens because men have frequent sex with their wives. They may force their wives to have sex with them.

See, different people have different levels of sexual desire. A man who has high desire won’t give any break (to his wife) and he will enjoy himself. (Male focus group).

As Muslims, if a man wants to have sex with his wife, whether she likes it or not, she must obey. If she doesn’t obey, she will be among the people on whom Allah’s wrath will fall. (Male focus group).

Participants in women’s groups blamed men for forcing sex on their wives.

It is the men that are lacking self-control. How will you deny a man his rights if he demands? No way at all. (Female focus group).

Let me tell you my sister, the husband is the chief and master of *kunika*. If he stops doing it, the woman won’t have *kunika*. (Female focus group).

#### Kunika is the will of god

Some women expressed the view that *kunika* was not something within their control.

*Kunika* is bad but if Allah gives you, there is nothing you can do. (Female focus group).

## Discussion

Men and women in our study had a clear understanding of the concept of *kunika.* They were unanimously of the view that *kunika* is a bad thing and described many adverse consequences. They went on to explain why *kunika* still happens, intentionally or otherwise.

In the Muslim, predominantly Hausa, communities in our study, many women and men defined *kunika* as a woman getting pregnant before weaning her previous child. This coincides with the advice of Islamic scholars that a woman should avoid pregnancy while breastfeeding [10]. While this is compatible with the WHO recommended inter-birth interval of 33 months [9], few participants in our study mentioned the number of months between pregnancies when explaining their understanding of *kunika*.

Some participants recommended a birth interval related to development milestones of the child, for example, the child should be walking before the mother becomes pregnant again. Other authors have described desirable birth interval in terms of the development of the child. The Havu people in the Kivu region of the Democratic Republic of Congo consider that a woman should not become pregnant again until the previous child has learned how to walk and fetch water from the lake, usually at about three years old [21, 22]. Among the Pokot people in Kenya, a woman should abstain from resuming sexual relations after giving birth, until the child is old enough to ‘go and fetch something’. [23] In both Kenya and Zimbabwe, this spacing of children might have arisen because it was easier for families to flee during wars and crises if only one child had to be carried [23, 24]. As in our study, women in a study in India said that a woman should wait to become pregnant again until the child starts walking and is able to do things on her own [25].

Quantitative studies have reported significant associations between short birth interval and adverse health consequences, particularly for the second child [5–8]. Participants in our focus groups recognized these direct health risks for the second child, but also stressed the risks for the mother’s health, which have been less apparent in quantitative studies [8]. In addition, they described indirect effects on the health and wellbeing of the immediately preceding child, of other siblings, and of the father. Quantitative studies have not examined these effects.

Men’s groups in our study described how *kunika* could lead to mental and physical health problems for men, including depression, hypertension and impotence. This is a new finding. Other studies on birth interval have not reported concerns about the effect of short birth interval on men’s health, possibly because many of them were limited to women participants [23, 24, 26]. When men did participate, the focus of discussions was on the implications of birth spacing on women’s health and well-being [3]. In conservative cultures, such as in Bauchi State, men are usually the main decision makers in a household, including decisions about the number and spacing of children. Discussing the health problems for men associated with *kunika* could be a useful way to draw men into the debate when seeking ways to reduce *kunika*.

Participants in our study recognized adverse consequences of *kunika* going beyond health. They described a crippling economic burden on the family: other children experience neglect and couples experience conflicts and stress as a result of limited resources. Men and women in North West Nigeria and India also stressed the economic burden imposed by short birth intervals [4, 25]. This economic hardship may be part of the reason for the effects of *kunika* on child nutrition and mortality [5, 6].

The social taboo associated with short birth interval described by participants in Bauchi is common in other settings. Carael reported that a woman who has short birth spacing is called *Kulikiza* (lit. Lazy woman) among the Havus [21], while Ferry reported that communities in Senegal use the word *Neffe* (meaning misfortune on the Wolof language) for a woman who becomes pregnant before she has weaned the previous child [26].

Despite the recognized disadvantages of *kunika*, it remains common in Bauchi. Participants in our study explained why some people choose *kunika* despite the risks: it builds larger families and the additional children can be a useful source of help and later of income. A study on child spacing attitudes in North West Nigeria reported that people choose short birth interval because rural communities consider children an asset because they work in the farm and provide help around the house [11]. In Kenya, a traditional community group endorsed short birth interval because it means more children in the family and children are a source of pride [23]. Some men in our focus groups also alluded to a large family as being something to be proud of. Another reason for choosing *kunika* mentioned in our study was competition between co-wives; this is important in a society where polygamy is common. Dean reports from a study in Kenya that competition between co-wives may decrease birth intervals [23].

According to our participants, *kunika* also happens inadvertently. They described it as a consequence of frequent (unprotected) sex, and linked this with women’s inability to refuse sex with their husbands. In a study on attitudes of Nigerian men regarding reproduction, men and women agreed that it is men who mostly decide when to initiate sex, the duration of abstinence from sex, and the number of children a couple should have [27]. Some women in our study were fatalist about short birth spacing. Similarly, some women among the Bani-lju tribe, part of the Havus living on mainland west of Lake Kivu, do not think that it is possible to avoid or to delay a pregnancy because conception is preordained by God [22].

### Strengths and limitations

Our findings are context-specific; they reflect what communities in Toro LGA, in Bauchi State, Nigeria, know and think about *kunika*, but we cannot necessarily generalize these findings, even to other similar settings, and we cannot assume that people in other settings share these views about birth spacing. Translating from the Hausa language to English before thematic analysis of the group reports may have lost or reduced meaning.

The expressed views of ordinary women and men confirmed that they consider *kunika* a bad thing, with many adverse effects. We have subsequently explored with community members their knowledge about causes and prevention of *kunika* as part of an effort to co-design ways to reduce this concern [28].

## Conclusions

Women and men in communities in Toro LGA, Bauchi State, have a clear perception of what is meant by *kunika*. Their rich understanding of its adverse consequences goes well beyond the relatively narrow health concerns reported in quantitative studies.

Including men in the discussions revealed their concerns about effects of *kunika* on men’s health and wellbeing. In the Bauchi context men continue to dominate decision-making about reproductive health; the findings from these focus groups will help us to promote male involvement in seeking ways to reduce *kunika*.

## Supplementary information

**Additional file 1.** Focus group guide.

## Data Availability

The data used and/or analyzed for the current study are included within the article. Additional information is available from the corresponding author on reasonable request.
