# Supplementary material for: “Kunika women are always sick”: views from community focus groups on short birth interval (kunika) in Bauchi state, northern Nigeria
Source: BMC Womens Health. 2020 May 24;20:113. doi: 10.1186/s12905-020-00970-2 (PMC7245922; doi:10.1186/s12905-020-00970-2)
Supplement: Supplementary file 1 — Additional file 1. Focus group guide. [file 12905_2020_970_MOESM1_ESM.pdf]

Focus Group Discussions (FGD) for Kunika

Assalam-o-Alaikum, my name is ________________________, and this is my colleague _______________. We work with FOMWAN and CIET, who are working together with the Bauchi State government and Toro LGA. We have been working in your community for the past two years on maternal and child health. We have heard from people in communities and from health service providers about their worries on Kunika. Today we are here to hear from you and discuss your views about Kunika. Your knowledge, experience and views can help the Local Government to understand this issue and plan better health services in the LGA.

Our discussion today is confidential. My colleague will take some notes on our discussion today so that we can report about your opinions, but we are not writing any names, so you can speak freely. Your participation today is entirely voluntary. If at any point you want to leave the discussion, you are free to do so. When reporting the discussion, we won't identify individual people, or even communities. We would also like to request all of you not to share anything expressed in the group with anyone outside this group. Our discussion will probably take about an hour. Thank you in advance for your time and for sharing your knowledge and views with us. May I start?

Ice breaker: **(ten minutes)**

1. So first, I would like to hear from each of you what do you think Kunika is? (Do a round, starting from one end and ask from each one).

summarise what the participants said and then proceed with section 2).

Section 2: Advantages of Kunika **(15 minutes)**

2. What do you think could be the advantages of Kunika?

2a. What could be the advantages for the family?

2b. What could be the advantages for men?

2c. What could be the advantages for the women?

2d. What could be the advantages for children?

2e. What could be the beneficial effects of Kunika on health especially of women and children

Section 3: Disdvantages of Kunika **(15 minutes)**

3. What do you think could be the disadvantages of Kunika?

3a. What could be the disadvantages for the family?

3b. What could be the disadvantages for men?

3c. What could be the disadvantages for the women?

3d. What could be the disadvantages for children?

2e. What could be the harmful effects of Kunika on health especially of women and children

Section 4: Overall group conclusion about Kunika **(ten minutes)**

4. Based on what we discussed so far about advantages and disadvantages of Kunika, overall, do you think Kunika is a good thing or bad thing?

4a. In what way is it overall a good thing?

4b. In what way is it overall a bad thing?

Section 5: Communicating on Kunika **(15 minutes)**

5. What would be a good way to inform men and women about disadvantages of Kunika?

5a (probe): If I were a woman/man who believed Kunika is a good thing and if you were to convince me that Kunica is bad, what would you say to me?

5b (probe): Who should say this to women/men like you?
